# Changes in the Dog’s and Cat’s Behaviors, as Reported by the Owners, before and during the Lockdown in China

**DOI:** 10.3390/ani12192596

**Published:** 2022-09-28

**Authors:** Sara Platto, Agathe Serres, Simona Normando, Yanqing Wang, Dennis C. Turner

**Affiliations:** 1 Department of Biotechnology, College of Life Sciences, Jianghan University, Wuhan 430056, China; 2 Institute of Deep Sea Science and Engineering, Chinese Academy of Sciences, 28 Luhuitou Road, Jiyang District, Sanya 572000, China; 3 Department of Comparative Biomedicine and Food Science, University of Padua, 35020 Padua, Italy; 4 China Conservation and Green Development Foundation, Beijing 110108, China; 5 Institute for applied Ethology and Animal Psychology, I.E.A.P./I.E.T., 8810 Horgen, Switzerland

**Keywords:** Wuhan lockdown, pets’ behavioral problems, cats during the lockdown, dogs during the lockdown, pets during the lockdown in China

## Abstract

**Simple Summary:**

It becomes critical to understand the effects that the current pandemic can have on the welfare of household dogs and cats, in order to develop programs that can support the owners in the care of their companion animals during such a critical time. The current survey showed that the constant presence of the owners at home during the lockdown favored the decrease in most of the behavioral problems considered. For example, during the lockdown period, dogs were more willing to play inside the house, while cats were more social and had a better appetite. In addition, litter box management for cats was improved during the lockdown, which might explain the decrease in the frequency of inappropriate elimination. Only anxiety-related behaviors in dogs increased during the lockdown, and these were associated with a reduction in play activity and altered sleeping patterns. On the other hand, most of the behavioral issues considered in the study were more frequent during the pre-lockdown period, which might have been associated with the long hours that pets spent alone at home, with reduced possibilities of interaction with their owners. Therefore, it is critical to develop support programs for pet owners, for both emergency situations such as lockdowns and normal times, to allow the establishment and maintenance of a healthy human–pet relationship and good pet welfare.

**Abstract:**

Considering the effects that the COVID-19 pandemic had and still has on human psychological health, it is expected that it might also affect household dogs’ and cats’ welfare. The current study explores the behavioral changes in dogs and cats before (BL) and during the lockdown (DL), as reported by their owners in China. Besides demographic parameters, variables related to the daily management of dogs and cats were analyzed in relation to behavioral problems, stress-related behaviors, and anxiety-related behaviors before and during the lockdown. A total of 261 questionnaires were collected. In general, behavioral problems and stress-related behaviors in dogs (*p* < 0.001) and cats (*p* < 0.001) decreased DL compared to BL, while anxiety-related behaviors in cats did not show any differences between the two periods considered. On the other hand, anxiety-related behaviors were more frequent in dogs DL (36.3%) compared to BL (35%), which were associated with reduced frequency of play activities with the owners (*p* = 0.016) and altered sleeping habits (*p* < 0.01). During the lockdown, dogs’ and cats’ daily routines and management (feeding and sleeping habits, dogs’ walks, dogs’ and cats’ play activities, litter box management, and cats’ lifestyle) experienced changes, but they were not associated with any behavioral issues. On the other hand, the behavioral issues considered for dogs and cats were more frequent BL, which were influenced by the daily management of the pets. The current study showed how critical the attention the owners can provide to the pets could be, to improve their companion animals’ welfare. Therefore, it is important to provide pet owners with behavioral management support both during particularly difficult periods such as a lockdown and during regular daily routines.

## 1. Introduction

It has been more than two years since the first COVID-19 lockdown in China, which started on 23 January 2020, yet, under the Chinese epidemic prevention and control legislation, cities in the country have been in and out of different levels of restrictions. These range from strict (people were not allowed to leave their homes), to medium (only one person per family was allowed to go out every three days for shopping), and flexible (people were allowed to go out for a few walks and for shopping with no limitations on the number of times per week), depending on the number of COVID-19 cases [1]. The COVID-19 pandemic restrictions have caused economic and social burden in all households across the world [2,3,4,5], including China [6], which did not exclude pet owners [7]. In fact, while pets provided certain comfort to their owners during the lockdown, the great stress caused by the restrictions on the household family members might have also affected the welfare of companion animals [8,9,10,11].

Studies have observed that the effects of the COVID-19 pandemic on pets’ welfare were different depending on the country of residence and type of pet. For example, an increase in the time spent by the owners walking and playing with their pets was reported during the lockdown, with the respondents perceiving the quality of life of their animals to be improved [12,13,14,15,16,17]. On the other hand, other reports found that the modification of the daily routine caused by COVID-19 restrictions increased the frequency of stress-related behaviors in dogs [8,9,10,11]. In Chinese cities under strict lockdown, dog walks were limited to once a day within the perimeter of the residential compound, while, in extreme cases, pets were not allowed to have access to outdoor areas. In this case, dog owners had to resort to other ways to allow their animals to relieve themselves, by using building rooftops or their bathrooms [1]. The decrease in the number of walks registered during the beginning of the pandemic was also a consequence of owners’ fear that their animals could get infected with SARS-Cov-2 virus [8,9]. A relationship between the ability of the owners to cope with the situation, their perception of a decrease in their own quality of life and their pets’ quality of life, and the frequency of behavioral problems in pets was found [18]. In fact, it has been recognized that when the quality of life of pet owners decreases, pets are 1.3 times more likely to poorly cope with the lockdown and to develop behavioral issues [8,9,18].

The forced cohabitation with their owners, a lack of exercise, and difficulties to find quiet time have also caused a disturbance of dogs’ physiological functions, such as eating and sleeping habits. For example, dog owners reported an increase in daytime sleepiness, sleep disturbances, and restlessness during the lockdown [19,20]. In general, dogs follow a diurnal circadian rhythm, with an activity pattern during the daylight hours, and sleep primarily during the night [21]. In general, the daily activity cycle of companion animals is under the complete control of their owners, including the time allocated to their pets [22]. It is well-known that daily exercise in dogs through walks reduces the incidence of behavioral problems, allowing the animal to release built-up energy [23]. Therefore, if dogs experience inactivity during the daytime, there might also be effects on their sleeping patterns [24,25,26]. During the lockdown, sleeping patterns were also altered in people who experienced wakefulness during the night because of daytime inactivity and stress [27,28]. This situation could also have disrupted the regular sleeping habits of dogs, in particular for those individuals who sleep in the same room as their owners (human–dog co-sleeping situations) [26]. In general, sleeping behaviors can also reflect an animal’s adaptation to its environment and can be used as indicators of welfare [29]. In fact, the relationship between sleeping habits and welfare is circular, with conditions that affect welfare also impacting sleep quality, while deterioration in sleep quality may have negative effects on animal welfare [29,30].

During the lockdown, there was a general concern among dog owners that their pets could become overweight because of the lack of exercise [17]. In addition, changes in the daily routine and the availability of pets’ food supplies during the lockdown might also have affected gastrointestinal functionality such as appetite and excretion in dogs [31]. For example, variation in food consumption in pets such as overeating or under-eating can be strongly affected by chronic and acute stress [32] or boredom [33].

The shutdown of business and education activities during the COVID-19 restrictions has resulted in children and parents spending a lot of time at home with their pets [34]. This forced cohabitation might have created an over-stimulated environment for the companion animals, which might have favored the development of an over-reactivity of dogs towards family members, in particular children [9]. In fact, a threefold increase in dog bites was reported in hospitals during the introduction of the COVID-19 restrictions in the USA [35], as well as in Italy and the UK, with dogs being more stressed and, therefore, more likely to lash out at people [15,34,36].

On the other hand, the current pandemic seems to have had positive effects on the life of household cats, with owners noticing their cats being calmer, more often seeking attention, being more playful, and exhibiting better appetite [9,37]. Despite these results, it is well-known that cats thrive in a consistent and predictable environment, where they can control the level and intensity of their interaction with their owners [38]. Therefore, the COVID-19 restrictions might have removed the ability of the cats to control the interaction with the household humans, which is a critical factor for felines’ welfare [38]. It has been noted that the reported low frequency of behavioral problems in cats during the lockdown might also be related to the inability of their owners to recognize typical stress-behaviors in their feline companions [38]). For example, cats under stressful conditions will tend to reduce the frequency and intensity of certain behaviors, which might be less obvious to the owners [39]. Therefore, many cats might attempt to reduce human exposure by either exiting the house for short periods of time or by hiding in places within the house [38]. Bowen et al. [9] reported that more than half of the surveyed cats were already kept indoors at the time of the lockdown, with a small percentage being used to having access to outdoor areas before the restrictions. Therefore, the forced cohabitation with the owners might have impaired the welfare of certain cats unable to ‘escape’ the suddenly intensified social environment, which could have triggered coping mechanisms in more sensitive individuals and the development of behaviors that were not well-accepted by the owners [38]. For example, Bowen et al. [9] found a positive correlation between cats’ not coping with the lockdown and the total number of behavioral problems getting worse, the increased owners’ closeness, and the owners’ concerns about the COVID-19 restrictions. Therefore, if cats do not have opportunities to become resilient to an intensified social life with the availability of ready access to hiding places, with the location of the resources away from children and adult activities, it is very unlikely that felines will be able to cope with the increased household social pressure [38]. Thus, it is possible that many cats would develop anxiety-related issues that can range from constant hiding to increased aggressive behaviors towards family members, or an increase in the frequency of previous behavioral problems such as urine marking or inappropriate elimination [38]. In addition, there is the possibility that once these behaviors are established, it is highly likely that these coping mechanisms will be retained within the cats’ behavioral repertoire after the restrictions end [38].

While many surveys have been performed across the world to assess the possible implications of the COVID-19 restrictions on the pets’ welfare [9,10,11,12,17,18,31,37,40], no studies have yet assessed the effects of the lockdown on dogs and cats in China. Therefore, the current study aims to assess the possible behavioral changes in dogs and cats before and during the lockdown, as reported by their owners in China. Two possible hypotheses will be considered in this study: (1) the behavioral issues of dogs and cats will increase during the lockdown because of the sudden changes of the daily routine and the constant presence of the household family members at home; and (2) dogs and cats will show a decreased frequency of behavioral issues because of increased attention received by the owners during the lockdown compared to before.

## 2. Materials and Methods

### 2.1. Survey Design

The first section of the questionnaire focused on the demographics of the respondents such as gender, age, education, job type before and during lockdown, family members affected by COVID-19, number of family members in the house before and during the lockdown, and type of lockdown they experienced during the pandemic (strict lockdown—not allowed to go out of the house; medium—people can only go out to buy food; flexible—people can go out for shopping and walks a few times a week). Additionally, the respondents were asked the type of pet owned (dog, cat, both), their characteristics (sex, age, breed, weight, number of owners—previous or current - persons responsible for the pet in the household or attachment figures for the pet), if there were additional pets besides the one surveyed, if the surveyed pet had a medical condition before (BL) or during the lockdown (DL), and the difficulties in finding pet supplies and veterinary services before and during the lockdown. Further, information regarding the management of the pets and the list of possible behavioral issues considered were also included. The behavioral issues assessed in the current study were grouped into three categories, behavioral problems, stress-related behaviors, and anxiety-related behaviors, each including a specific set of behaviors related to the period before and during the lockdown (Table 1). The rest of the questionnaire was structured with two scales: a modified version of Companion Animal Bonding Scale (CABS) from Poresky et al. [40], and the Perceived Stress Scale (PSS) from Roberti et al. [41] (for details and scale analysis, see Platto et al. [1]).

### 2.2. Survey Method

The questionnaire and survey were approved by the Research Department of Jianghan University on June 2020 (Wuhan, China). The Chinese and English versions of the questionnaire were uploaded to the online platform Wèn Juàn Xīng (Changsha, Ranxing Information Technology Co., Ltd, China), which produced a weblink and a QR code that were sent to the public around China through the main Chinese social media platforms (WeChat, Weibo). The questionnaire was addressed to people who owned dogs or cats or both. The respondents were able to access the questionnaire by using their phones, computers, or tablets. The uploaded questionnaire had an initial part that explained the purpose of the survey and the anonymity of the responses. Pet owners with multiple animals of the same species in the house were recommended to choose only one of them for the survey. In addition, during the uploading of the questionnaire to the online platform, we selected the option that did not allow partially filled-out questionnaires to be submitted. As the entire questionnaire was rather long and took time to complete (approximately 30 min), the reply rate for this convenience sample was rather low. The link remained open from 1 July 2020 till 30 June 2021, allowing respondents to complete the survey at their own convenience.

### 2.3. Statistical Analysis

All statistical analyses were performed using R 3.6.0 (R core team, 2020). Responses for cats and dogs were analyzed separately. First, the responses to questions concerning animals’ behavioral problems, stress-related behaviors, and anxiety-related behaviors were transformed into ordinal data (never = 1, always = 5). Responses were then summed within each category to obtain a single variable for each behavioral issue category labeled “behavioral problems”, “stress-related behaviors”, and “anxiety-related behaviors”. 

Regarding the animals’ living condition variables, the food type variable was not included in the analysis for cats, since all owners used cat food. For number of family members, three categories were created: 1–2 family members, 3–4 family members, or 5+ family members. For sleeping habits, the categories “sleeps all day and awake occasionally at night” and “sleeps more during the day” for dogs and “awake at night and sleeps more during the day” and “sleeps virtually all day, awake occasionally at night” for cats were merged. The time spent playing, the feeding frequency, and the frequency of litter refilling and scooping were analyzed as ordinal variables. For cats’ personality, “limited social” and “social with some members of the family” categories and “fearful” and “hiding all the time” were merged.

The effect of the animals’ living conditions on exhibited behavioral problems, stress-related behaviors, and anxiety-related behaviors was analyzed using linear mixed effect models (LMMs) with the “lmer()” function from the “lme4” package [43]. Three models were fitted for each species, with response variables being one of the three behavioral issues. Predictors included the number of family members, living situation, frequency of walks, frequency of feeding, food type, feeding habits, sleeping habits, time spent playing, medical condition, personality, and lockdown type and period for dogs. Predictors included the number of family members, living situation, feeding habits, sleeping habits, number of litter boxes, frequency of litter brand change, frequency of litter refilling, frequency of litter scooping, time spent playing, medical condition, personality, and lockdown type and period for cats (see Table 2). An interaction between lockdown type and period was included in models, and the animal’s ID, breed, sex, and age category were added as random factors. 

A detailed analysis was conducted for aggressive and biting events for dogs and aggressive behaviors and spraying for cats. The association between these behavioral problems and other variables was analyzed using one-way repeated ordinal regressions (“clmm()” from the “ordinal” package [44]). Two models were run for each species (one per behavioral problem). Variables included in these models as predictors were number of family members, lockdown type, period, frequency of stress-related behaviors, and frequency of anxiety-related behaviors for dogs and frequency of litter brand change, number of family members, lockdown type, period, frequency of stress-related behaviors, and frequency of anxiety-related behaviors for cats.

A model diagnosis was conducted to check LMMs, including normality of residuals and homogeneity of variances. Multi-colinearity was also checked using a variance inflection factor (VIF), with no major issues (no VIF > 3). Wald chi-squared tests were used to obtain *p*-values from all models. Pairwise tests were conducted by running the same models with appropriate sub-settings and applying a Bonferroni correction (for additional information please refer to the “Supplementary Material”).

## 3. Results

A total of 261 completed questionnaires were received. Overall, 44.4% of the respondents underwent a strict lockdown, 23.4% a medium one, and 32.2% a flexible one, with 41.8% of the respondents coming from the city of Wuhan and 58.2% coming from other cities around China.

### 3.1. Dogs

During the lockdown, changes in the daily lives of dogs were recorded. Although some differences were relatively small and not statistically significant, they still indicate the direction of changes. For example, while the once-a-day walk did not change before (25.6%) compared to during the lockdown (25.6%), a decrease in the walks taken twice a day (DL: 32.1%; BL: 50%) and three or more times a day (DL: 12%; BL: 16.7%) was reported. In addition, during the lockdown, there was an increase in both dogs that could only gain access to a garden (17.9%) and dogs that were never walked but could only stay inside the house (8.9%), compared to before the lockdown (only garden: 2.4%; only inside house: 5.4%). Play activities also showed changes between the two time periods considered. For example, an increase in short-time play was recorded during the lockdown (10 min/day: 15.5%; 30 min/day: 26.2%) compared to before (10 min/day: 6.5%%; 30 min/day: 23.2%), with a decrease in the 1 h/day play (DL: 36.3%; BL: 47.6%). Dogs’ living situation (being able to move freely within the house, being kept inside a cage or on the terrace) did not show any changes before compared to during the lockdown. Moreover, dogs willing to play inside the house increased during the lockdown (29.8%) compared to before (22%), as well as very calm/sleepy dogs (BL: 9.5%; DL: 11.3%), while active dogs were more frequent before the lockdown (68.5%), compared to during the COVID-19 restrictions (58.9%). Dogs’ physiological activities such as sleeping and feeding habits also showed some modifications during the two periods considered. Precisely, dogs awake at night were more frequent during the lockdown (53%) compared to before (47%), while dogs that slept sometimes during the day/were sometimes awake at night (6.5%) and dogs active during the day/sleeping at night (46.5%) were more frequent before the lockdown, compared to during the COVID-19 restrictions (dogs active during the day/sleeping at night: 5.3%; dogs active during the day/sleeping at night: 41.7%). Dogs’ appetite also showed variations with individuals with finicky appetite increasing during the lockdown (28%), compared to before (23.8%), while dogs with good appetite decreased (BL: 61.9%; DL: 57.7%). Dogs with voracious appetites did not show any differences between the two periods considered. The variation of appetite might also be caused by modifications of the diet during the lockdown. Indeed, dog owners used slightly more canned dry food before the lockdown (94.6%) compared to during the COVID-19 restriction (92.3%%), while the use of table scraps slightly increase during the lockdown (BL: 5.4%; DL: 7.7%). Medical conditions were more frequent before the lockdown (9.5%), compared to during the COVID-19 restrictions (7.7%).

#### 3.1.1. Behavioral Problems (BPs)

**Figure 1:**
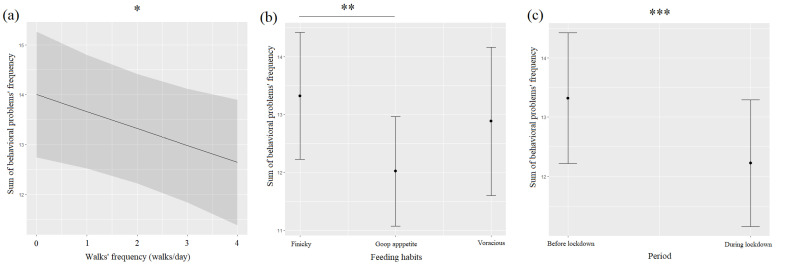
Means and 95% confidence intervals of dogs’ behavioral problems frequency depending on walks frequency (**a**), dogs’ feeding habits (**b**), and period (**c**) extracted from linear mixed-effect models (LMMs). *: *p* < 0.05; **: *p* < 0.01; ***: *p* < 0.001, Wald chi-squared tests with Bonferroni correction.

Behavioral problems (BPs) were more frequent before (36.6%) than during (27.2%) the lockdown, and they were significantly associated with the pre-lockdown period (Table 3, Figure 1c). A detailed analysis of BPs showed that submissive behaviors (57.1%), fear of people (45.2%), excessive excitability/impulse control (44.1%), house soiling (feces and urine) (41.7%), destructive behaviors (39.9%), and excessive barking (37.5%) were more frequent before than during the lockdown (overly submissive behaviors: 52.4%; fear of people: 36.3%; excessive excitability/impulse control: 28.6%; house soiling: 38.7%; destructive behaviors: 32.7%; excessive barking: 26.2%), while aggressive behaviors/biting (18%) and howling (15%) were slightly higher during the lockdown compared to before (howling: 13%; aggressive behaviors/biting: 15%). BPs were negatively associated with the number of walks (Table 3, Figure 1a) and significantly linked to feeding habits (Table 3, Figure 1b): dogs with finicky appetite showed more behavioral problems than dogs with good appetite (*p* = 0.001).

#### 3.1.2. Stress-Related Behaviors (SRBs)

**Figure 2:**
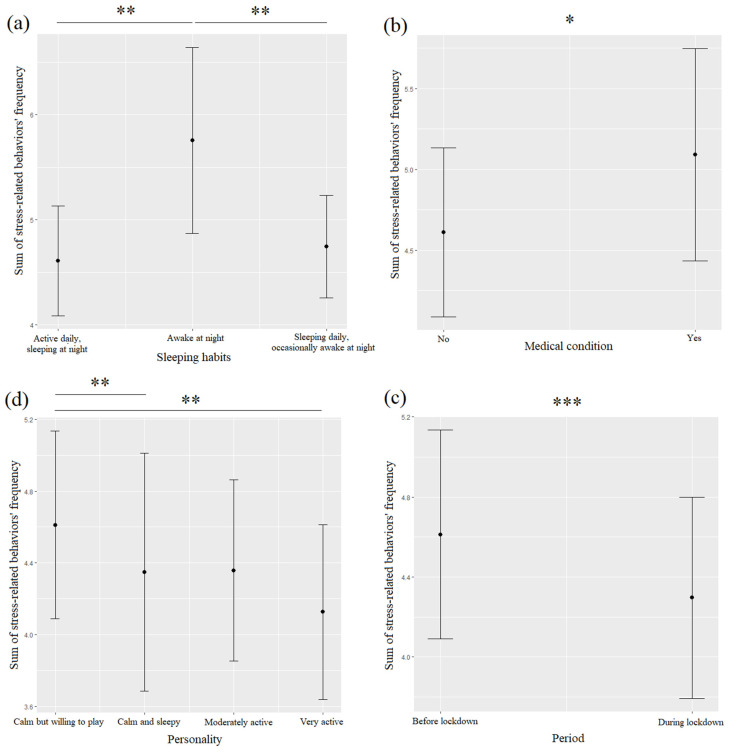
Means and 95% confidence intervals of dogs’ stress-related behaviors’ frequency depending on dogs’ sleeping habits (**a**), medical condition (**b**), personality (**c**), and the period (**d**) extracted from linear mixed-effect models (LMMs). *: *p* < 0.05; **: *p* < 0.01; ***: *p* < 0.001, Wald chi-squared tests with Bonferroni correction.

**Figure 3:**
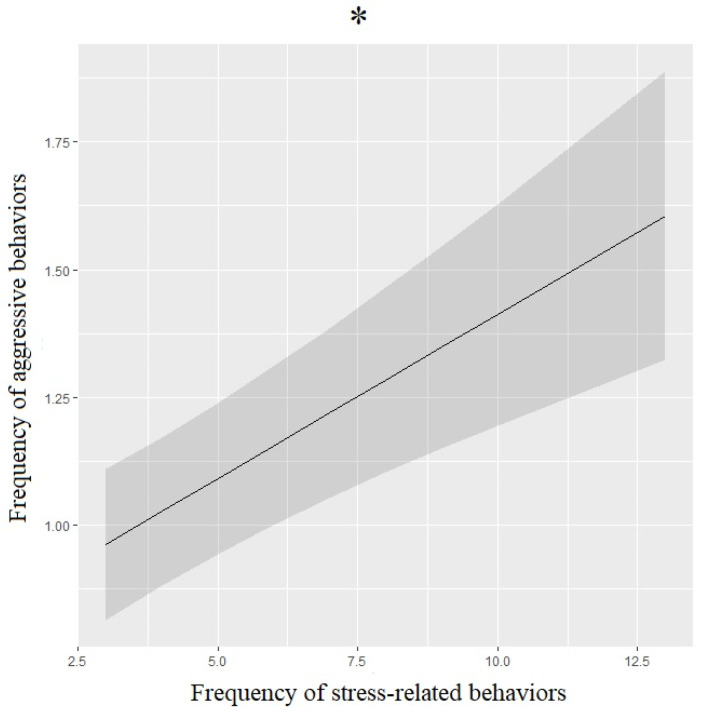
Means and 95% confidence intervals of dogs’ aggressive behaviors’ frequency depending on the frequency of stress-related behaviors extracted from ordinal logistic regression. *: *p* < 0.05, Wald chi-squared test.

Stress-related behaviors (SRBs)were more frequent before (31.7%) than during (26.2%%) the lockdown, and they were significantly associated with the pre-lockdown period (Table 3, Figure 2c). A detailed analysis of SRBs showed that hiding (32.1%), intense shedding (36.9%), and visible dandruff (26.2%) were more frequent before the lockdown than during it (hiding: 23.8%; intense shedding: 32.1%; visible dandruff: 22.6%). SRBs were significantly linked to sleeping habits (Table 3, Figure 2a): dogs that were awake at night exhibited significantly more stress-related behaviors than dogs that were active during the day and sleeping at night (*p* = 0.003) or sleeping during the day and occasionally awake at night (*p* = 0.004). Dogs that were suffering from medical conditions also exhibited significantly more SRBs (Table 3, Figure 2b). The exhibited SRBs were significantly linked to the dogs’ personality (Table 3, Figure 2d): dogs that were calm but willing to play exhibited significantly more stress-related behaviors than dogs that were calm and sleepy (*p* = 0.005) or very active (*p* = 0.003). Dogs that exhibited more SRBs were also more aggressive towards family members (Table 4, Figure 3).

#### 3.1.3. Anxiety-Related Behaviors (ARBs)

**Figure 4:**
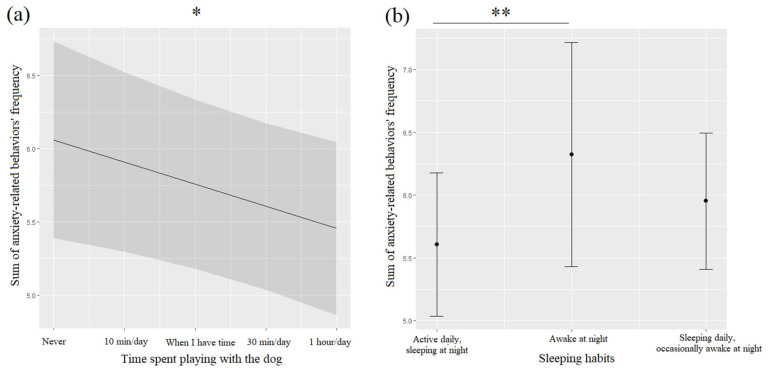
Means and 95% confidence intervals of dogs’ anxiety-related behaviors’ frequency depending on the time spent by owners to play with dogs (**a**) and dogs’ sleeping habits (**b**) extracted from linear mixed-effect models (LMMs). *: *p* < 0.05; **: *p* < 0.01, Wald chi-squared tests with Bonferroni correction (significant results are bold: *p* < 0.05).

Anxiety-related behaviors (ARBs) were not significantly associated with either of the two time periods considered, even though they showed a slightly higher frequency during the lockdown (36.3%), compared to before (35%). A detailed analysis of ARBs showed that episodes of yawing (40.5%) and episodes of nose licking (45.8%) were more frequent before the lockdown than during it (episodes of yawing: 38.7%; episodes of nose licking: 42.9%), while episodes of grooming (45.3%) and episodes of panting/body tense (18.5%) were more frequent during the lockdown than before it (episode of grooming: 41.7%; episode of panting/body tense: 11.9%%). ARBs were negatively associated with the time owners spent playing with them (Table 3, Figure 4a), and they also were significantly linked with sleeping habits (Table 3, Figure 4b): dogs that were awake at night showed higher frequency of ARBs compared to dogs that were active during the day and sleeping at night (*p* = 0.010).

### 3.2. Cats

During the lockdown, changes in household cats’ lives were recorded. Although some differences were relatively small and not statistically significant, they still indicate the direction of changes. For example, 86.6% of cats were already indoor individuals before the lockdown, and the number increased to 92.1% during the lockdown, which was confirmed by the decreased number of cats allowed access to the outdoors (DL: 7.9%; BL: 13.4%). The number of cats kept in cages within the house also increased during the lockdown (11.8%) compared to before (8.7%), which was also supported by the decrease in the number of cats that could move freely in the house (DL: 88.2%; BL: 91.3%). The cats’ activities also showed changes between the two periods considered. For example, the short-time play activity (10 min/day) decreased during the lockdown (8.7%), compared to before (17.3%), while longer playtime (30 min/day) (53%) and the “*only when owner has time*” (35%) increased, compared to before the lockdown (30 min/day: 50%; “*only when owner has time*”: 30%). In addition, cats with irregular daytime sleep/erratic nocturnal patterns (11.8%), and sleep around the clock (13.4%) increased during the lockdown compared to before (irregular daytime sleep/erratic nocturnal patterns: 9.5%; slept around the clock: 11.8%), while cats that ’sleep more during the day’ or ‘awake at night’ were more common before the lockdown (BL: 78%; DL: 74.8%). On the other hand, feeding habits showed an increased good appetite during the lockdown (66.2%), compared to before (60.6%), while the finicky (23.6%) and voracious (10.2%) categories decreased compared to before the lockdown (finicky: 24.4%; voracious: 15%). Cats’ personality also showed slight changes, with an increase in cats being social during the lockdown (31.5%), compared to before (28.3%), while fearful cats (2.4%) and cats that were “limited social” (64%) decreased during the COVID-19 restriction compared to the pre-lockdown period (fearful cats: 3.2%; “limited social” cats: 69%). There was only a very small percentage of cats that were hiding all the time during the lockdown (0.1%). Litter box management showed an improvement during the lockdown. Precisely, litter changing (refill) performed twice a week increased during the lockdown (18.1%) compared to before (7.1%), while the one time a week refill decreased during the lockdown (47.2%), compared to before (51.2%). The once-a-month frequency refill did not show differences during the two time periods considered. Similarly, twice-a-day scooping (35.4%) and once-a-week scooping (16.5%) increased during the lockdown compared to before (twice a day: 33.1%; once a week: 15%), while once-a-day scooping decreased during the lockdown (DL: 48%; BL: 52.8%). In addition, the frequency of changes of litter brand was generally higher during the pre-lockdown period (one time: 23.6%; two times: 18.9%; more than three times: 19.7%) compared to during the lockdown (one time: 20.5%; two times: 14.2%; more than three times: 8.7%). The number of litter boxes did not really vary between the two time periods. There was only a slight increase in the one–two litter boxes during the lockdown (89%) compared to before (87.4%), while three–four and five and more litter boxes did not change at all (BL/DL.: three–four: 9.5%; five and more: 1.6%). Before the lockdown, there was only a small percentage of cats without litter box (1.6%). Surveyed owners in China did not change the food of the cats, but they kept using “*only canned dry food*” during both periods. The medical conditions in cats were slightly higher before the lockdown (15.7%) than during the restrictions (13.4%). 

#### 3.2.1. Behavioral Problems (BPs)

**Figure 5:**
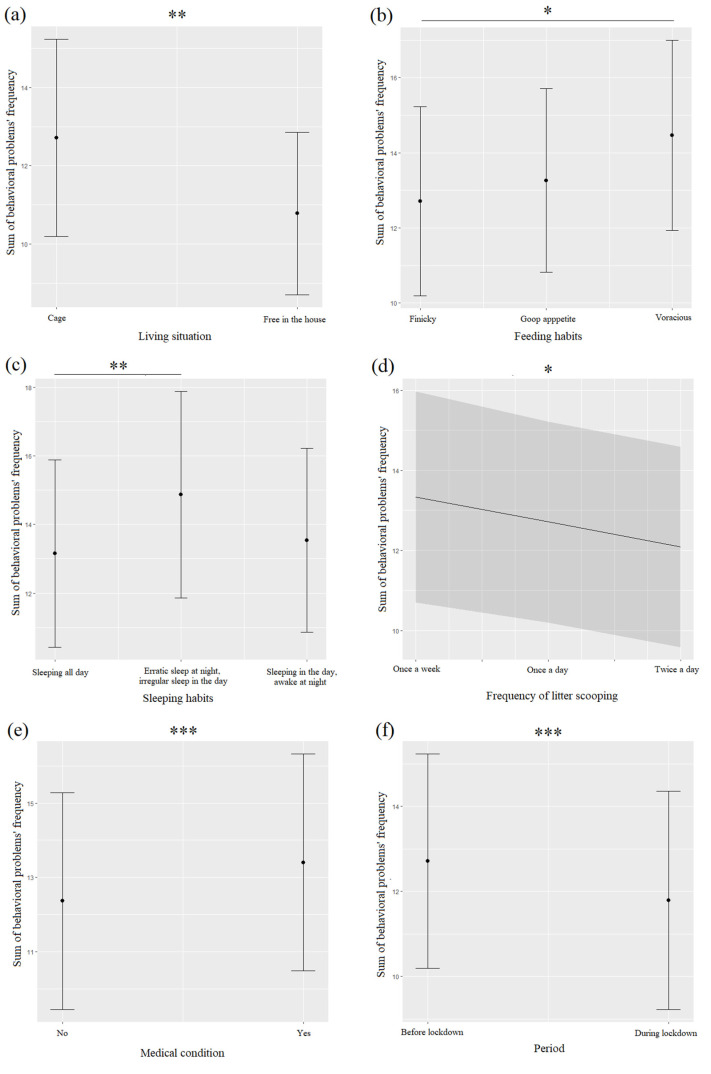
Means and 95% confidence intervals of cats’ behavioral problems frequency depending on cats’ living situation (**a**), feeding habits (**b**), sleeping habits (**c**), frequency of litter scooping (**d**), medical condition (**e**), and the period (**f**) extracted from linear mixed-effect models (LMMs). *: < 0.05; **: *p* < 0.01; ***: *p* < 0.001, Wald chi-squared tests with Bonferroni correction.

Behavioral problems (BPs) were more frequent before (37.1%) than during (34%) the lockdown, and they were significantly associated with the pre-lockdown period (Table 5, Figure 5f). A more detailed analysis showed inappropriate elimination (65.4%), destructive behaviors (60%), and aggressive/bites (19.7%) increased during the pre-lockdown period, compared to during the COVID-19 restrictions (inappropriate elimination: 49%; destructive behaviors: 52.8%; aggressive/bites: 18.1%). Only urine-spraying behavior showed an opposite trend, with an increased frequency during the lockdown (20%) compared to before (18.1%). The BPs were significantly more frequent for cats living in a cage than for those living free in the house (Table 5, Figure 5a). BPs’ frequency was linked with cats’ feeding and sleeping habits (Table 4): voracious cats exhibited more behavioral problems than finicky cats (*p* = 0.13, Figure 5b), while cats sleeping all day exhibited fewer behavioral problems than those with erratic nocturnal sleeping patterns and irregular day sleeping patterns (*p* = 0.006, Figure 5c). The frequency of behavioral problems was negatively linked with the frequency of litter scooping (Table 5, Figure 5d). This frequency was higher for cats suffering a medical condition than for those that did not (Figure 5e). 

#### 3.2.2. Stress-Related Behaviors (SRBs)

**Figure 6:**
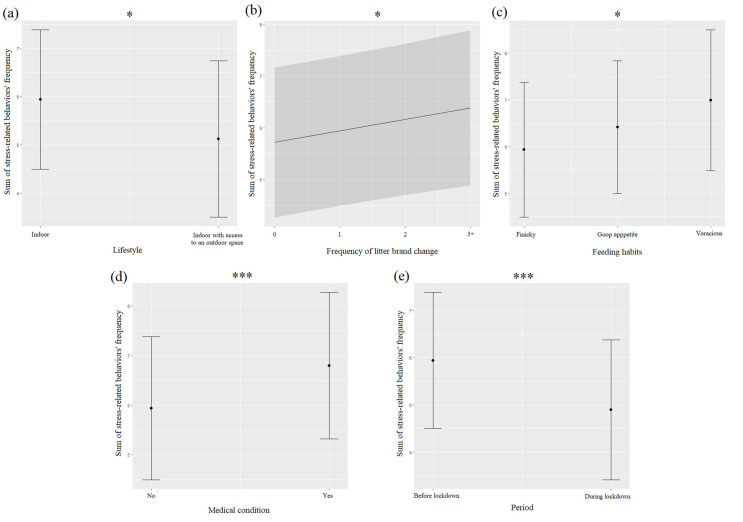
Means and 95% confidence intervals of cats’ stress-related behaviors’ frequency depending on cats’ lifestyle (**a**), frequency of litter brand change (**b**), feeding habits (**c**), medical condition (**d**), and the period (**e**) extracted from linear mixed-effect models (LMMs). *: *p* < 0.05; ***: *p* < 0.001, Wald chi-squared tests with Bonferroni correction.

Stress-related behaviors (SRBs)were less frequent during (33.6%) than before (49.3%) the lockdown, and they were significantly associated with the pre-lockdown period (Table 5, Figure 6e). A more detailed analysis showed that hiding (75.6%), intense shedding (51.2%), and visible dandruff (25.2%) were more frequent before than during the lockdown (hiding: 60%; intense shedding: 18%; visible dandruff: 22%). SRBs were significantly more frequent for cats living indoors than for those that had access to an outdoor space (Figure 6a) and when owners changed the litter brand more often (Table 5, Figure 6b). The feeding habits of the cats impacted the frequency of SRBs, but pairwise comparisons were not significant with a Bonferroni correction (Table 5, Figure 6c). The frequency of SRBs was higher for cats suffering a medical condition than for those that did not (Figure 5d). 

#### 3.2.3. Anxiety-Related Behaviors (ARBs)

**Figure 7:**
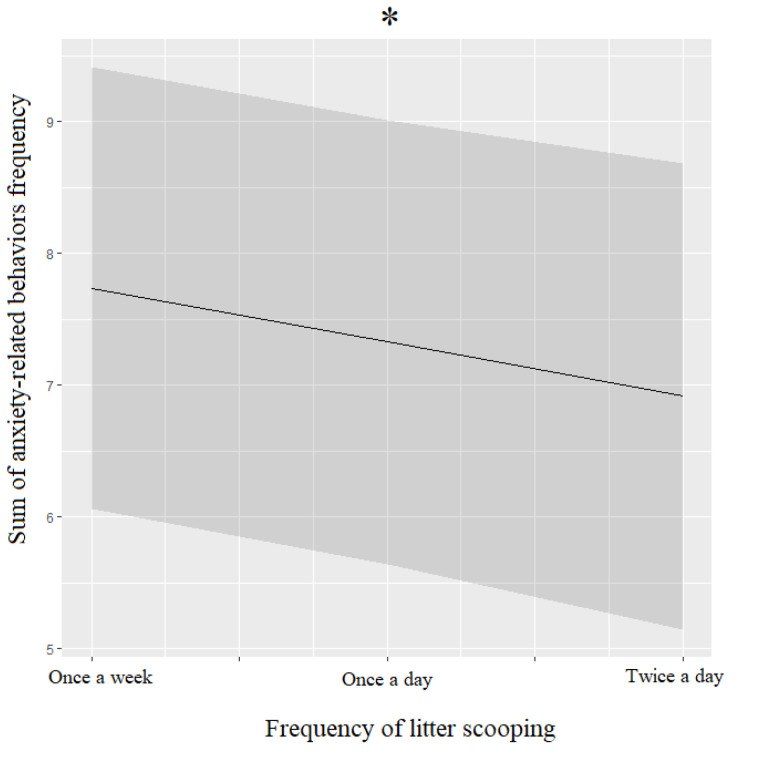
Means and 95% confidence intervals of cats’ anxiety-related behaviors’ frequency depending on the frequency of litter scooping extracted from linear mixed-effect model (LMM). *: *p* < 0.05, Wald chi-squared test.

Anxiety-related behaviors (ARBs) did not show significant differences between before and during the lockdown (BL: 42.2%; DL: 42.2%). Despite that, a more detailed analysis showed that episodes of grooming were more frequent before (58.3%) than during (50.4%) the lockdown, while episodes of howling (17.3%) and meowing (54.3%) were higher during the lockdown than before (episodes of howling: 12.6%; episodes of meowing: 49.6%). On the other hand, episodes of pacing around the house did not show any differences between the two periods considered (BL/DL: 48%). The ARBs’ frequency was negatively linked to the frequency of litter scooping (Table 5, Figure 7). 

**Figure 8:**
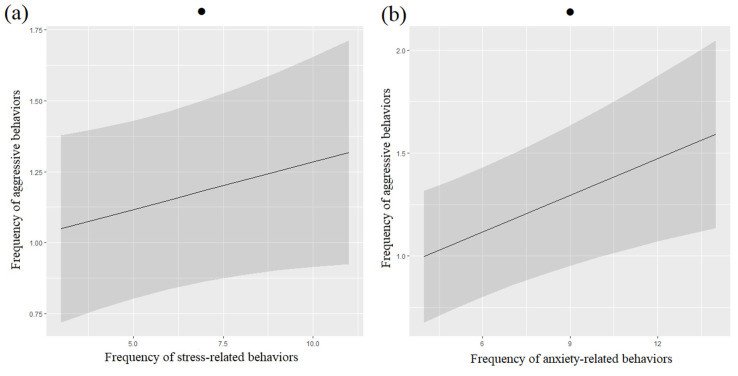
Means and 95% confidence intervals of cats’ aggressive behaviors’ frequency depending on the frequency of stress-related behaviors (**a**) and frequency of anxiety-related behaviors (**b**), extracted from ordinal logistic regressions. ●: *p* < 0.10, Wald chi-squared tests.

Aggressive behaviors towards family members tended to be positively associated with ARBs and SRBs in cats (Table 6, Figure 8). The number of family members also tended to be associated with cats’ aggressive behaviors, but pairwise tests were not significant.

**Figure 9:**
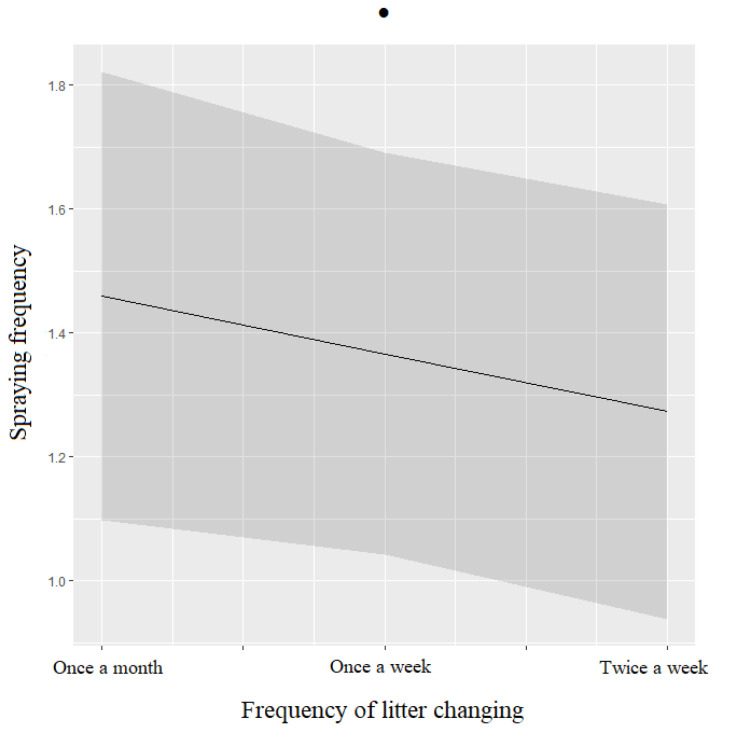
Means and 95% confidence intervals of cats’ spraying frequency depending on the frequency of litter changing, extracted from ordinal logistic regression: ●: *p* < 0.10, Wald chi-squared test.

Cats’ spraying tended to be negatively associated with frequency of litter changing (Table 5, Figure 9).

## 4. Discussion

Considering the great psychological impact that the COVID-19 pandemic has caused and is still causing on people, it was expected that this would also reflect on the pets’ welfare [9,18,45,46], depending on factors such as the pre-pandemic management of the animals, the country of origin/type of lockdown experienced, and how the owners coped with the situation [9,17,20,31]. The current study reported a decreased frequency of the behavioral problems and stress-related behaviors in dogs and cats during the lockdown compared to before, while anxiety-related behaviors in cats did not show significant differences during the two periods considered. On the other hands, household dogs in China showed a slightly increase in anxiety-related behaviors during the lockdown, which was associated with the reduced frequency of play activities with the owners and with the increase in dogs’ altered sleeping patterns. Overall, the lockdown in China has caused changes of the household pets’ daily routines such as a decreased frequency of the dogs’ daily walks, and an increase in cats kept in cages or not being allowed to gain access to the outdoors. In addition, the modification of cats’ management during the COVID-19 restrictions might have also caused an increased frequency of urine spraying in feline companions.

Three-quarters of the respondents in China spent between a few weeks and three months at home with their pets during the lockdown, which could have possible implications for the companion animals’ welfare. For example, in the present study, dogs’ behavior improved, with both behavioral problems and stress-related behaviors decreasing during the lockdown compared to before. This disagrees somewhat with what was found in studies carried out in other countries, which reported either no differences in behavioral complaints about pets [17,19] or a decrease in companion animals’ welfare [9]. The increased time spent at home by owners during the lockdown might have allowed them to pay more attention to the needs of their dogs during interactions and play activities [17,19], a possibility that might be supported by the fact that dogs’ willing to play inside the house increased in number during the lockdown in China. Differences in the routine care of pets, and in the ways people coped with restrictions worldwide may partly explain the different results we present for Chinese owners.

However, the frequency of anxiety-related behaviors increased in dogs during the lockdown compared to before, in particular howling and body tension/panting behaviors, and these might be an expression of dogs’ frustration in the absence of the usually performed outdoor activities [9]. This might also explain the association between the occurrence of anxiety-related behaviors and the frequency of play activities and the dogs’ sleeping habits. Indeed, surveyed pet owners in China reported a decrease in the time their dogs were engaged in physical activities, being the time dedicated to active play and the frequency of daily walks. For example, while the frequency of walks simply decreased, the pattern was more nuanced for play activities, with a decrease in long ones (i.e., 1 h/day) and an increase in shorter ones (i.e., 10 min/day, 30 min/day). Previous studies have also recorded an increase in dogs’ behavioral issues in relation to the frequency and duration of physical activities such as walks and playtime. Moreover, an increase in the number of dogs that could only gain access to a local private garden or that could never be taken outside was also found during the lockdown in China. It is important to remember that the number of dog walks allowed was subjected to limitations depending on the type of lockdown in China. Strict lockdown only allowed once-a-day walks within the perimeter of the residential compound, or, in extreme cases, dogs were not allowed access to the outdoors at all [1]. This situation might have increased dogs’ restlessness, barking, and anxiousness during the lockdown, as found also in other surveys [9,31,34,39].

The slight increase in lack of sleep at night, for dogs in China during the lockdown compared to before, is consistent with other studies reporting changes in dogs’ sleeping habits during COVID-19 restrictions [9,20,34,47]. This might be a consequence not only of changes in the daily routine of the companion animals but also of those of the owners’ sleeping habits [27], in particular if the dogs and the owners slept in the same room (human–dog co-sleeping) [26]. Altered sleeping patterns in animals can also suggest a deterioration of their welfare, especially since these patterns can reflect the ability of an individual to cope with the environment [29]. Boredom can also cause increased drowsiness during the day and sleep disruption [48], which is linked to behavioral problems [42].

Although other studies have found that owners feared their dogs were becoming overweight because of the lack of exercise during the COVID-19 restrictions [9,20], the number of dogs with finicky appetite increased in our sample, whereas the number of individuals with a good appetite decreased during the lockdown. This trend may be due to owners not being able to source the usual pet food due to the COVID-19 restrictions and the animals not appreciating the alternative. This result might be also supported by the fact that the number of owners using table scraps as food for the dogs slightly increased during the lockdown in China. In addition, the reduction in appetite could also be a response to the possibly overstimulating environment created by the constant presence of the family at home. The latter explanation might also be supported by the increase (although small) of aggressive behaviors/bites found during the lockdown in the surveyed dogs in China, which concurs with what was found in other studies [9,20,26,34,35]. In general, aggressive behaviors in dogs are characterized by underlying anxiety due to unpredictability in the social and physical environment [49]. Even though the lockdown in China might have brought some improvement in the dogs’ welfare because of the increased interaction with the owners, the changes in the daily routine still have brought some discomfort to the animals. Therefore, it would be important to establish online community services to support owners with the daily care of their dogs, in case of tight COVID-19 restrictions. For example, Esam et al [17] reported that New Zealand pet owners received online “pet behavioral support” during their lockdown, which was very effective in reducing pet relinquishment. 

On the other hand, most of the behavioral problems (submissive behaviors, fear of people, excessive excitability/impulse control, house soiling, destructive behaviors) and the stress-related behaviors (hiding, intense shedding, visible dandruff) investigated were associated with the pre-lockdown period, probably caused by the pets having to spend most of the day alone at home with fewer opportunities to interact with their owners. For example, stress-related behaviors were more frequent in dogs that were more willing to play inside the house. Indeed, due to the relation between stress response, social behaviors, and dogs’ personality [50], the abovementioned issue could have been perceived more severely by this type of dogs, especially if the walks were their only opportunity for daily interaction with their owners during the pre-lockdown period. The limited possibility of interactions with owners and, possibly, an under-stimulated house environment during the pre-lockdown period could have caused boredom and frustration, which, in turn, could lead to excessive barking, destructive behaviors, and even aggressive incidents [42]. This could explain the association between aggressive behaviors in dogs during the pre-lockdown period and the higher frequency of stress-related behaviors found in the present study. Yang et al. [51] found a higher risk of dog bites in China for dogs that spent less time interacting with their owners, had fewer exercise opportunities, and were left alone longer. In addition, lack of stimulation and boredom might also have altered the sleeping patterns of the dogs. Indeed, there was a higher frequency of stress-related behaviors in dogs awake at night during the pre-lockdown periods. In general, the circadian rhythms in household dogs are under the complete control of their owners [22], and the dogs’ rest being interrupted by multiple periods of casual activity throughout dark hours might disrupt their sleep patterns [23,52,53,54] and impact their welfare.

Moreover, the higher frequency of behavioral issues and stress-related behaviors before the lockdown could also be caused by some individuals being affected by separation anxiety disorders, when alone in the house or missing a reference figure. In general, these disorders are characterized by excessive barking [55], destructive behaviors, excessive excitability/impulse control, and intense shedding [42]. In addition, excessive dandruff, when not caused by medical conditions, can be also linked to stress, including altered water and food intake in dogs [56], which causes decreased skin hydration [57]. Indeed, dogs under episodes of separation anxiety neglect food and water [56]. This could also explain the positive association between finicky appetite and frequency of behavioral problems in the current survey, during the period before the lockdown. Unfortunately, a specific assessment of separation anxiety in dogs was not performed in the present survey. Therefore, only a possible relation between the behavioral problems and stress-related behaviors mentioned above and the presence of separation anxiety in some of the surveyed dogs in China can be hypothesized. Furthermore, the increased presence of dandruff in dogs might also be related to the presence of medical conditions, which were more frequent during the pre-lockdown period and were also associated with stress-related behaviors. The relationship between behavioral issues and medical conditions can be complex. For example, musculoskeletal pain and painful gastro-intestinal and dermatological conditions are commonly recognized as having a significant influence on animal-behavior problems [58]. 

Overall, the results of the current study concerning the pre-lockdown period underscore the importance of providing dogs with a stimulating environment, for example with pet-sitting services when available, in order to avoid the emergence of behavioral issues. In China, there are a few companies (Spare Leash, Petbacker, Moi Pet) that offer such services, using dog-experienced volunteers who bring dogs to parks and play with them. However, these services are mainly limited to the biggest cities such as Shanghai, Beijing, and Guangzhou [59]. Therefore, more active services in the local communities and through veterinary hospitals should be developed, in order to offer dog owners support in the daily care of their companion animals. In addition, China does not yet have a well-developed network of veterinary behaviorists, which makes it even more difficult for owners to be supported in case their dogs have behavioral issues, and this, unfortunately, favors the relinquishment of pets.

The pet owners surveyed in China also reported a decrease in behavioral issues in cats during the lockdown, with an overall reduction in the frequency of behavioral problems (urine spraying, inappropriate elimination, destructive behaviors, aggressive behaviors) and stress-related behaviors (hiding, intense shedding, visible dandruff), while anxiety-related behaviors (episodes of grooming, episode of howling, episode of meowing, pacing) did not show differences during the two periods considered. This unexpected improvement in cats’ behaviors during the COVID-19 restrictions could have been influenced by the increased time spent at home by the owners, who might have provided better attention towards feline management and needs [38]. In fact, the current study also reported an increase in the number of cats being more social during the lockdown and a reduction in fearful or less social individuals. On the other hand, it is also possible that the low frequency of behavioral issues detected in cats during the lockdown in China might also be caused by the inability of the owners to recognize signs of distress in their cats, which often are characterized by a decrease in frequency and intensity of certain behaviors [38]. Yet, some changes in cats’ management were reported during the lockdown, which might still have affected feline welfare, even though they were not associated with any of the investigated behavioral issues. For example, more cats were kept indoors during the lockdown, as already found in a similar study, probably because the owners were afraid their felines could get infected with SARS-Cov-2 [60]. In addition, an increased number of cats kept in cages within households was also reported, which corresponded to a decreased number of cats being allowed to move freely within the house. In general, in China, it is not unusual to see single or multiple cats kept in cages for part of the day, or permanently, independently of COVID-19 restrictions. Nevertheless, these changes in cats’ lifestyles probably created some discomfort for household felines, as indicated by the more detailed analysis of anxiety-related behaviors, where howling and meowing increased slightly during the lockdown. Further, cat owners also reported an increase in urine-spraying behavior during the lockdown, which was not related to litter box management during this period. In fact, litter box management showed an improvement during the lockdown compared to before, with an increased frequency of daily scooping (twice a day) and of litter refilling (twice a week), even though changes of litter brand decreased, probably caused by the suspension of the business activities for animals’ supplies in the Chinese cities with strict and medium lockdown. Improved management of the litter box might also explain the general reduction in inappropriate elimination observed in the cats in China during the lockdown. Therefore, the increased frequency of urine-spraying behavior found during the lockdown might be explained in two ways: (1) the owners might have become more aware of the urine-spraying behavior of their cats because they were spending more time at home with their feline companions during the lockdown [38]; or (2) cats that were not allowed to gain access to the outdoors or were being kept in cages during the lockdown might have performed urine spraying because they no longer had the opportunity to do this outside the house (or the cage). 

The changes of cats’ lifestyles during the lockdown and the constant presence of the household family members in the house might also have altered the resting patterns of felines. In fact, as it was previously observed for dogs, cats also showed an increased number of individuals presenting irregular daytime sleep/erratic nocturnal patterns or sleeping around the clock during the lockdown, compared to before. In general, cohabitation with humans is a factor that influences food intake and circadian rhythms in cats, with felines’ activity corresponding with the peak of human activity [61]). The constant presence of family members at home during the lockdown might have made it difficult for cats to find a quiet space and time for their daily napping. In general, adult cats spend an average of 13–18 h a day sleeping, divided into multiple naps during the day, which is very important for their welfare [62,63]. This might also explain the presence of a very small number of cats (0.1%) hiding all the time during the lockdown in China. In addition, cats kept in cages or not allowed to roam in the outdoors during the lockdown might have experienced a drastic reduction in physical activity, which might have led them to sleep throughout the day or to have an irregular sleeping pattern [38]. On the other hand, cats in China showed an increase in good appetite during the lockdown, which concurred with the study reported by Jezierski et al. [37]. It is possible that owners paid more attention to the general management of their felines, as happened for the litter box. Indeed, differently than for dogs, cat owners did not change food types during the lockdown and kept using “canned and dry food”. This might be explained by the fact that there is a well-developed online community among Chinese and foreign cat owners in China, through social media such as WeChat. These cat-owner communities have shown, at different times, that they have a great ability for owners to support each other during the lockdown, for practical matters such as delivering pet food to people in need or even recovering a pet, in the case of when the owners were taken to the hospital because of a COVID-19 infection. The same cannot be said for dogs.

As previously mentioned for dogs, most of the behavioral issues assessed in cats in the current study were also associated with the period before the lockdown, with poor litter management having an impact on them. For example, a lower frequency of litter scooping was associated with an increased occurrence of anxiety-related behaviors and behavioral problems in cats, while the number of times the owners changed the litter brand was associated with a higher frequency of stress-related behaviors. Urine-spraying behavior in cats was also associated with the frequency of litter refilling in the pre-lockdown period. In general, cats have different thresholds of tolerance for poor litter box management, depending on the surrounding environment, so anxiety-provoking events or the presence of underlying stressors can alter this threshold [38,64,65,66,67,68]. Indeed, urine spraying is considered a sign of elevated arousal that can be caused by social and environmental stressors [69,70,71], as shown by elevated fecal glucocorticoids found in cat sprayers compared to cats that showed inappropriate elimination [72]. The cats surveyed in China showed a slightly higher frequency of pacing around the house before the lockdown, which is considered a typical behavior of cat sprayers and indicates a high level of arousal in these individuals [73]. Moreover, unlike cat sprayers, individuals with inappropriate elimination are at higher risk of having an increased frequency of medical problems [74]. As mentioned earlier, inappropriate elimination was more frequent in cats before the lockdown, and this might be related to possible underlying medical conditions. Indeed, the current study reported a slightly higher presence of medical conditions in cats before the lockdown. It is well-known that a history of lower urinary tract disease in cats has also been found to increase the likelihood of house-soiling problems by nearly fourfold [73]. Data from urinary tract diseases have shown that these medical issues could also be relevant to cats that present spraying behaviors [74]. 

Moreover, it is possible that, as mentioned for dogs, cats surveyed in China might have experienced long hours alone at home during the pre-lockdown period with limited interaction with their owners. This might explain the higher frequency of stress-related behavior in indoor cats and the presence of behavioral problems in cats kept in cages most of the day. In general, cats have retained a strong need for opportunities to engage in predatory activities, either for hunting outdoors or for performing exploratory/chasing behaviors after a toy in the house [39]. Thus, preventing felines from performing these behaviors might be detrimental to their welfare [39] and might also lead to the development of boredom [75,76]. The latter might also lead cats to develop destructive behaviors such as chewing and scratching, which were more frequent before the lockdown. These behaviors, even though they are considered normal for outdoor cats, could become unpleasant for the owners when performed inside the house [77]). Further, confined spaces such as cages, besides limiting the cats’ species-specific behaviors, reduce the animals’ avoidance of unwanted interactions from family members, thus increasing the felines’ stress levels [77,78]. In general, the first mechanism of cats to avoid interaction with humans would be escaping and hiding [79,80], but if these possibilities are limited or nonexistent, the animals will resort to aggression [38]. This is supported by the findings of the current study, where aggressive behaviors/bites were somewhat higher in cats before the lockdown, and they were positively associated with anxiety-related behaviors and negatively associated with the number of family members in the house. It was interesting to notice that in the current study there was a decrease of 4.2% in the number of family members in the household and an increase of 3.8% in the pet owners living alone during the lockdown, compared to before. This might be explained by the fact that, during the lockdown in China, different families decided to separate their members, for example, with children going to live with their grandparents and parents staying in a different house, in order to reduce the risk of COVID-19 contagion [1]. Normally, household families in China are usually composed of parents, one child (or two), and one or two grandparents who take care of the grandchildren. Thus, a possibly crowded pre-lockdown environment might have been stressful for cats, and the enhanced exposure to family members and the range of activities that cats are required to do by their owners might trigger defensive mechanisms such as biting and scratching [81]. Indeed, owners in China reported a higher number of cats being less social and more fearful during the period before the lockdown. This might support the hypothesis of a more stressful environment for cats before the lockdown, which was also characterized by the increased frequency of hiding and shedding. In general, when felines are stressed, their muscles get tense and the follicles of some hairs (referred to as telogen hairs) are released with consequential loss of coat [42]. 

Moreover, a stressed individual might also show altered sleeping and feeding habits. Owners surveyed in China reported a higher frequency of behavioral problems and stress-related behaviors in cats that showed erratic day sleeping/awakening at night and voracious appetite. Boredom could also play an important role in these altered physiological activities [33], where feeding time might be perceived by the cats as the only enriched moment of the day [76]. Therefore, owners should be informed of the necessity to give cats areas where they can withdraw to avoid unwanted attention sites where cats can easily access their resources such as food, water, and litter, without disturbances from family members, and sites where cats can perform their regular sleeping habits [38]. Moreover, one way to enrich the time animals spend on feeding behavior is to place small amounts of dry food into containers with holes, through which cats have to extract the individual pieces [82,83], or by hiding small pieces of food in the surrounding house environment to make feeding more interesting. Toy-like objects that are destructible and have nutritional value may be of interest to cats, but there are few such items available commercially [84]. 

In general, it is important to provide cats with a balanced social and physical environment, where they can express normal feline-like behaviors, and access to perches or hanging areas, where they can hide or avoid undesired human interactions or have their regular daily naps [42]. In addition, a variety of toys, replaced regularly, should be available to cats, whereby quality and texture must be carefully considered in order to elicit play or predatory behaviors. Objects that are mobile, have complex surface textures, and mimic prey characteristics are the most successful in promoting play in cats [85]. Finally, human interaction should also be part of the environment. Regular periods of time, which are not part of care-taking procedures (feeding), should be available every day, for cats to interact with their owners [86]. In fact, the more owners respond to their cats, the more likely the cats are to respond to them, and interactions initiated by cats last longer than those initiated by owners [86]. 

## 5. Limitations of the Study

This study faced a low response rate, despite the numerous posts on Chinese social media and the support of the survey by a broad network of animal protection organizations in China. The survey was available online for one year, since cities in China have experienced different waves of lockdown from the beginning of 2020 until now. The low response rate might be explained by the fact that the questionnaire was probably too long, which discouraged many people from participating, thus limiting the sample size. In addition, the survey was performed several months after the first lockdown in China was lifted (Wuhan lockdown, 23 January–8 April 2020). Therefore, some respondents had to reply to the statements on the questionnaire retrospectively, which might have influenced the results. Nevertheless, the current study represents the first assessment of the comparison of behavioral issues in dogs and cats in China, before and during the lockdown. Therefore, the findings still can add valuable information to the existing worldwide literature on the subject.

## 6. Conclusions

The present study found that the lockdown may have had overall positive effects on Chinese pets (for dogs: more willing to play and fewer behavioral problems and stress-related behaviors compared to before the lockdown; for cats: better appetite, more social, and improved litter box management by owners), probably due to the presence of the owners at home. However, some negative effects were also found, such as anxiety-related behaviors in dogs occurring more often as well as reduced frequency of play activities with the owners and altered sleeping habits. Some changes in the lifestyle of the pets, which might have influenced their welfare, were also found (for dogs: reduced frequency of dog walks and play activities with their owners, possibly leading to altered sleeping and eating patterns; for cats: increased number of indoor cats and individuals kept in cages, possibly leading to increased urine spraying and disruption of sleeping habits). The overall general improvement found could be consistent with the owners being busy with their daily routine and the pets being alone at home for most of the day, prior to lockdown. These findings, even though inconclusive, still highlight two important points. (1) When restrictions on the movements of people and their pets are instated, it would be critical to develop support tools for pet owners such as online behavioral consultations, as previously successfully developed in New Zealand [17], which might also reduce animal relinquishment. In addition, the existing and well-developed Chinese community neighborhood support, which was very effective during the lockdown for other emergencies, could also be used to support households with pets. (2) It is concerning that pets may have spent most of the day alone at home, with reduced interaction with their owners (limited to the time of walks and feeding), prior to lockdown. China lacks a well-developed net of veterinary behaviorists, who could support pet owners in case of behavioral problems in their dogs and cats. Therefore, it is important to develop courses for veterinarians focused on appropriate dog and cat behavioral management, in order to help owners in developing a healthy relationship with their companion animals. 

## Figures and Tables

**Table 1:** List of variables related to the management of the household dogs (a) and cats (b) and the list of behavioral issues, each assessed BL and DL.

**(a) Dog’s Medical Conditions**	**Specified by the Owner**
Dog’s living situation	Free to move in the house Kept in a crate for most or part of the day Kept in the garden with chances to move inside the house Kept on a terrace with chances to move inside the house
Dog’s sleeping habits	Sleeps more during the day/occasionally awakens at night Awake all night Active during the day/sleep at night
Dog’s feeding habits	Finicky, good appetite, voracious
Dog’s play activity	1 h/day, 30 min/day, 10 min/day, only when have time
Behavioral problems [42]	House soiling (urination/defecation) Excessive barking Howling Destructiveness (chewing furniture, scratching, digging) Overly submissive behavior Excessive excitability/impulse control deficit Aggressive towards family members/biting Fear of people
Stress-related behaviors [42]	Hiding Intense shedding Visible dandruff
Anxiety-related behaviors [42]	Episodes of yawing Episodes of nose licking Episodes of grooming Episodes of panting/body tense
**(b) Cat’s Lifestyle**	**Indoor Only** **Partially Indoor/Partially Outdoor**
Cat’s medical conditions	Specified by the owner
Cat’s living situation	Free to move in the house Kept in cage and let out few hours a day
Cat’s sleeping habits	Sleeps during the day/awake at night Erratic nocturnal pattern/irregular daytime pattern Sleeps around the clock
Number of litter boxes	0, 1–2, 3–4, 5+
Changes of litter brand (number of times)	0, 1, 2, 3+
Litter refilling	Once a week, twice a week, once a month
Scooping litter	Once a day, twice a day, once a week
Cat’s feeding habits	Finicky, good appetite, voracious
Cat’s play activity	30 min/day, 10 min/day, only when have time
Cat’s personality (before and during lockdown)	Social Limited social Fearful Hiding all the time
Behavioral problems [42]	Urine spraying Inappropriate elimination (feces/urine) Destructive behaviors (chewing, scratching) Aggressive behaviors/biting
Stress-related behaviors [42]	Hiding Intense shedding Visible dandruff
Anxiety-related behaviors [42]	Episodes of yawing Episodes of howling Episodes of meowing Episodes of pacing around the house

**Table 2:** Variables included in linear mixed-effects models (LMMs) for dogs (a) and cats (b).

**(a) Dogs**	**Variables**	**Levels**
**Response variables**	Behavioral problems	–
	Stress-related behaviors	–
	Anxiety-related behaviors	–
**Predictors**	Family members	1–2/3–4/≥5
	Living situation	Cage/free in the house/garden/terrace
	Walks frequency	0–4 times a day
	Feeding frequency	Once a day/twice a day/anytime
	Food type	Dog food/table scraps
	Feeding habits	Finicky/good appetite/voracious
	Sleeping habits	Active daily, sleeping at night/sleeping daily, occasionally awake at night/awake at night
	Time spent playing	Never/10 min a day/when have time/30 min a day/1 h a day
	Medical condition	Yes/no
	Personality	Very calm/calm but willing to play/moderately active/very active
	Lockdown type	Flexible/medium/strict
	Period	Before lockdown/during lockdown
**Random factors**	Individual	Individual ID
	Breed	Individual breed
	Sex	Female/male
	Age	Puppy/adolescent/adult/senior
**(b) Cats**		
**Response variables**	Behavioral problems	–
	Stress-related behaviors	–
	Anxiety-related behaviors	–
**Predictors**	Family members	1–2/2–3/≥5
	Living situation	Cage with opportunity to go out occasionally/free in the house
	Lifestyle	Indoor/indoor and outdoor
	Feeding habits	Finicky/good appetite/voracious
	Sleeping habits	Sleeping around the clock/sleeping daily, occasionally awake at night/eratic nocturnal pattern and irregular daytime pattern
	Number of litter boxes	1–5
	Frequency of litter brand change	0–3+
	Frequency of litter refilling	Twice a week/once a week/once a month
	Frequency of litter scooping	Twice a day/once a day/once a week
	Time spent playing	Never/10 min a day/when have time/30 min a day/1 h a day
	Medical condition	Yes/no
	Personality	Fearful/limited social/social
	Lockdown type	Flexible/medium/strict
	Period	Before lockdown/during lockdown
**Random factors**	Individual	Individual ID
	Breed	Individual breed
	Sex	Female/male
	Age	Kitten/junior/prime/mature/geriatric

**Table 3:** Statistical outputs from linear mixed-effects models (LMMs) for dogs. Significant results (*p* < 0.05) are bold.

Response Variable		Behavioral Problems	Stress-Related Behaviors	Anxiety-Related Behaviors
**Predictors**	Family members	χ² = 2.86, df = 2, *p* = 0.505	χ² = 1.33, df = 2, *p* = 0.536	χ² = 1.24, df = 2, *p* = 0.455
	Living situation	χ² = 2.93, df = 3, *p* = 0.402	χ² = 3.04, df = 3, *p* = 0.386	χ² = 1.54, df = 3, *p* = 0.672
	Walks frequency	**χ² = 4.93, df = 1,** ***p* = 0.026**	χ² = 0.23, df = 1, *p* = 0.634	χ² = 0.30, df = 1, *p* = 0.586
	Feeding frequency	χ² = 0.04, df = 1, *p* = 0.837	χ² = 0.06, df = 1, *p* = 0.796	χ² = 0.99, df = 1, *p* = 0.320
	Food type	χ² = 1.75, df = 1, *p* = 0.186	χ² = 1.44, df = 1, *p* = 0.230	χ² = 2.26, df = 1, *p* = 0.133
	Feeding habits	**χ² = 10.27, df = 2,** ***p* = 0.006**	χ² = 4.60, df = 2, *p* = 0.099	χ² = 1.62, df = 2, *p* = 0.443
	Sleeping habits	χ² = 3.15, df = 2, *p* = 0.207	**χ² = 6.25, df = 2,** ***p* = 0.0438**	χ² = 4.64, df = 2, *p* = 0.098
	Time spent playing	χ² = 0.04, df = 1, *p* = 0.838	χ² = 0.96, df = 1, *p* = 0.326	**χ² = 5.83, df = 1,** ***p* = 0.016**
	Medical condition	χ² = 0.74, df = 1, *p* = 0.391	**χ² = 4.02, df = 1,** ***p* = 0.045**	χ² = 0.04, df = 1, *p* = 0.845
	Personality	χ² = 4.09, df = 3, *p* = 0.252	χ² = 5.99, df = 3, *p* = 0.112	χ² = 2.30, df = 3, *p* = 0.513
	Lockdown type	χ² = 0.68, df = 2, *p* = 0.712	χ² = 2.01, df = 2, *p* = 0.367	χ² = 0.01, df = 2, *p* = 0.997
	Period	**χ² = 26.63, df = 1,** ***p* < 0.001**	**χ² = 13.44, df = 1,** ***p* < 0.001**	χ² = 0.05, df = 1, *p* = 0.832
	Lockdown type * period	χ² = 2.93, df = 3, *p* = 0.402	χ² = 1.21, df = 2, *p* = 0.547	χ² = 1.16, df = 2, *p* = 0.560

* Correlation between lockdown type and period (before and during lockdown).

**Table 4:** Statistical outputs from ordinal logistic regressions for dogs.

Response Variable		Aggressive Behaviors	Bites
**Predictors**	Family members	χ² = 0.92, df = 2, *p* = 0.631	χ² = 0.58, df = 2, *p* = 0.748
	Lockdown type	χ² = 0.13, df = 2, *p* = 0.936	χ² = 0.14, df = 2, *p* = 0.933
	Period	χ² = 0.22, df = 1, *p* = 0.643	χ² = 2.61, df = 1, *p* = 0.106
	Stress-related behaviors	**χ² = 4.64, df = 1,** ***p* = 0.031**	χ² = 1.19, df = 1, *p* = 0.274
	Anxiety-related behaviors	χ² = 0.84, df = 1, *p* = 0.360	χ² = 1.30, df = 1, *p* = 0.253

**Table 5:** Statistical outputs from linear mixed-effects models (LMMs) for cats (significant results (*p* < 0.05) are in bold).

Response Variable		Behavioral Problems	Stress-Related Behaviors	Anxiety-Related Behaviors
**Predictors**	Family members	χ² = 1.75, df = 2, *p* = 0.525	χ² = 1.23, df = 2, *p* = 0.359	χ² = 0.86, df = 2, *p* = 0.477
	Living situation	**χ² = 7.58, df = 1,** ***p* = 0.006**	χ² = 0.88, df = 1, *p* = 0.348	χ² = 3.36, df = 1, *p* = 0.066
	Lifestyle	χ² = 0.87, df = 1, *p* = 0.351	**χ² = 4.93, df = 1,** ***p* = 0.026**	χ² = 1.02, df = 1, *p* = 0.311
	Feeding habits	χ² = 5.95, df = 2, *p* = 0.051	**χ² = 6.89, df = 2,** ***p* = 0.032**	χ² = 5.79, df = 2, *p* = 0.055
	Sleeping habits	χ² = 4.83, df = 2, *p* = 0.089	χ² = 2.19, df = 2, *p* = 0.334	χ² = 0.21, df = 2, *p* = 0.89
	Number of litter boxes	χ² = 0.01, df = 1, *p* = 0.912	χ² = 0.01, df = 1, *p* = 0.911	χ² = 0.44, df = 1, *p* = 0.506
	Frequency of litter brand change	χ² = 0.69, df = 1, *p* = 0.406	**χ² = 5.77, df = 1,** ***p* = 0.016**	χ² = 0.49, df = 1, *p* = 0.484
	Frequency of litter refilling	χ² = 0.87, df = 1, *p* = 0.351	χ² = 0.03, df = 1, *p* = 0.869	χ² = 2.68, df = 1, *p* = 0.101
	Frequency of litter scooping	**χ² = 5.10, df = 1,** ***p* = 0.024**	χ² = 0.03, df = 1, *p* = 0.859	**χ² = 5.20, df = 1,** ***p* = 0.022**
	Time spent playing	χ² = 2.23, df = 1, *p* = 0.136	χ² = 0.00, df = 1, *p* = 0.973	χ² = 1.76, df = 1, *p* = 0.184
	Medical condition	**χ² = 11.08, df = 1,** ***p* < 0.001**	**χ² = 11.25, df = 1,** ***p* < 0.001**	χ² = 0.83, df = 1, *p* = 0.362
	Personality	χ² = 2.89, df = 2, *p* = 0.235	χ² = 5.20, df = 2, *p* = 0.074	χ² = 0.09, df = 2, *p* = 0.955
	Lockdown type	χ² = 0.87, df = 2, *p* = 0.648	χ² = 3.06, df = 2, *p* = 0.216	χ² = 0.09, df = 2, *p* = 0.952
	Period	**χ² = 24.73, df = 1,** ***p* < 0.001**	**χ² = 38.33, df = 1,** ***p* < 0.001**	χ² = 0.12, df = 1, *p* = 0.730
	Lockdown type * period	χ² = 1.46, df = 2, *p* = 0.483	χ² = 1.08, df = 2, *p* = 0.583	χ² = 3.29, df = 2, *p* = 0.193

* Correlation between lockdown type and period (before and during lockdown).

**Table 6:** Statistical outputs from ordinal logistic regressions for cats.

Response Variable		Aggressive Behaviors	Bites
**Predictors**	Family members	χ² = 5.66, df = 2, *p* = 0.069	χ² = 0.90, df = 2, *p* = 0.956
	Frequency of litter change	χ² = 0.14, df = 1, *p* = 0.709	χ² = 3.29, df = 1, *p* = 0.069
	Lockdown type	χ² = 0.37, df = 2, *p* = 0.829	χ² = 0.06, df = 2, *p* = 0.967
	Period	χ² = 2.61, df = 1, *p* = 0.106	χ² = 0.02, df = 1, *p* = 0.902
	Stress-related behaviors	χ² = 3.19, df = 1, *p* = 0.074	χ² = 2.47, df = 1, *p* = 0.116
	Anxiety-related behaviors	χ² = 3.66, df = 1, *p* = 0.055	χ² = 0.23, df = 1, *p* = 0.628

## Data Availability

Original data can be available upon personal request to the authors.

## Supplementary Materials

The following supporting information can be downloaded at: https://www.mdpi.com/article/10.3390/ani12192596/s1, File S1: R script for the statistical analysis of the changes in the dogs and cats behavior before and during the lockdown in China.
